# Crystal structure of 1,3-bis­(4-methyl­benz­yl)-1*H*-1,3-benzimidazol-3-ium bromide monohydrate

**DOI:** 10.1107/S2056989014025857

**Published:** 2015-01-01

**Authors:** Sevim Türktekin Çelikesir, Ömer Çelik, Senem Akkoç, İlhan Özer İlhan, Yetkin Gök, Mehmet Akkurt

**Affiliations:** a Department of Physics, Faculty of Sciences, Erciyes University, 38039 Kayseri, Turkey; b Department of Physics, Faculty of Education, Dicle University, 21280, Diyarbakir, Turkey, and, Science and Technology Application and Research Center, Dicle University, 21280, Diyarbakir, Turkey; c Department of Chemistry, Faculty of Sciences, Erciyes University, 38039 Kayseri, Turkey; d Department of Chemistry, Faculty of Arts and Sciences, İnönü University, 44280 Malatya, Turkey

**Keywords:** crystal structure, 1,3-bis­(4-methyl­benz­yl)-1*H*-3,1-benzimidazol-3-ium bromide monohydrate, benzimidazolium salts, N-heterocyclic carbenes, hydrogen bonds, aromatic π–π stacking inter­actions

## Abstract

In the title hydrated symetrically substituted 1,3-bis­(4-methyl­benz­yl)benzimidazolium salt, C_23_H_23_N_2_^+^·Br^−^·H_2_O, the dihedral angles between the benzimidazole ring system (r.m.s. deviation = 0.003 Å) and the pendant benzene rings are 73.18 (16) and 77.52 (16)°. Both benzene rings lie to the same side of the benzimidazole ring system, giving the cation an overall U-shape. In the crystal, the cation is linked to the water mol­ecule by a short C—H⋯O hydrogen bond and the water mol­ecule forms O—H⋯Br hydrogen bonds. Together, these inter­actions lead to [010] chains. The packing is consolidated by C—H⋯Br hydrogen bonds and aromatic π–π stacking inter­actions [centroid–centroid distances = 3.5401 (17) and 3.8815 (18) Å], generating a three-dimensional network.

## Related literature   

For general background to N-heterocyclic carbenes (NHCs), which have been frequently used as ligands in organometallic and coordination chemistry, see: Arduengo *et al.* (1991 ▸); Akkoç & Gök (2013 ▸); Akkoç *et al.* (2014 ▸); Berding *et al.* (2009 ▸); Gök *et al.* (2014 ▸). For related structures, see: Akkurt *et al.* (2011 ▸, 2012 ▸).
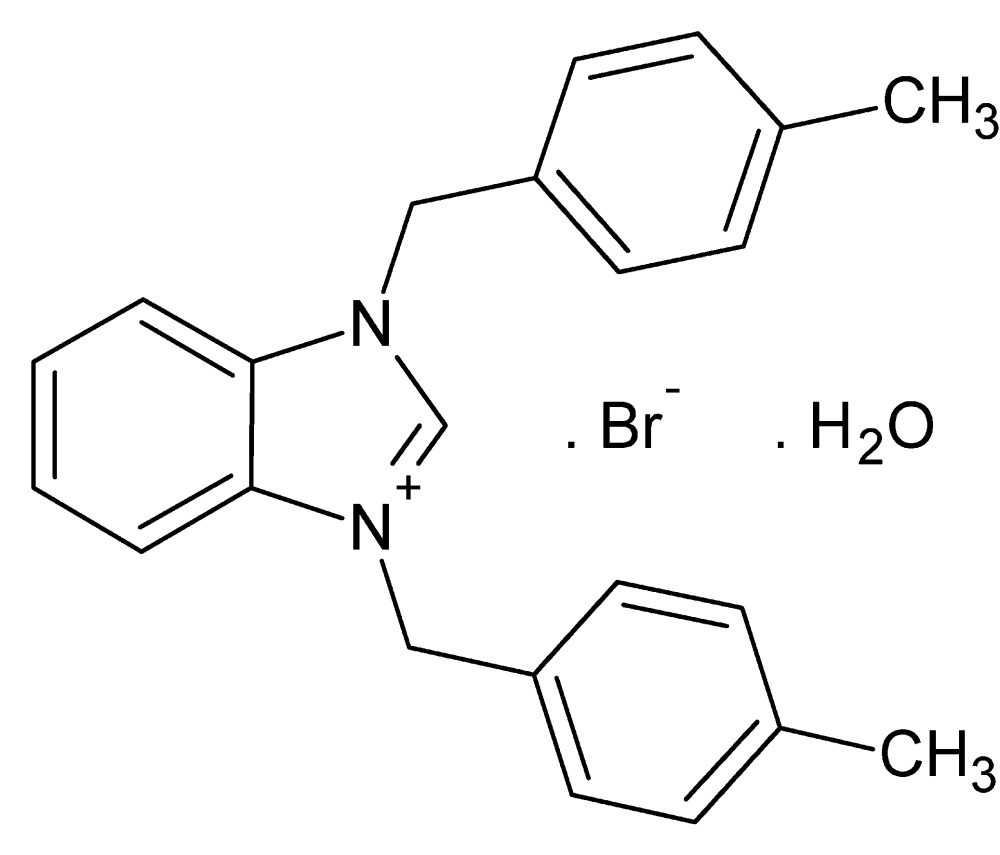


## Experimental   

### Crystal data  

C_23_H_23_N_2_^+^·Br^−^·H_2_O
*M* *_r_* = 425.35
Triclinic, 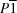

*a* = 9.3846 (3) Å
*b* = 9.7174 (3) Å
*c* = 12.5603 (4) Å
α = 76.405 (2)°
β = 84.739 (2)°
γ = 72.696 (2)°
*V* = 1062.65 (6) Å^3^
*Z* = 2
Mo *K*α radiation
μ = 1.95 mm^−1^
*T* = 296 K
0.15 × 0.10 × 0.06 mm

### Data collection  

Bruker APEXII CCD diffractometer
22302 measured reflections
4342 independent reflections
3144 reflections with *I* > 2σ(*I*)
*R*_int_ = 0.035

### Refinement  

*R*[*F* ^2^ > 2σ(*F* ^2^)] = 0.048
*wR*(*F* ^2^) = 0.135
*S* = 1.06
4342 reflections
252 parameters
2 restraints
H atoms treated by a mixture of independent and constrained refinement
Δρ_max_ = 1.15 e Å^−3^
Δρ_min_ = −0.49 e Å^−3^

### 

Data collection: *APEX2* (Bruker, 2007 ▸); cell refinement: *SAINT* (Bruker, 2007 ▸); data reduction: *SAINT*; program(s) used to solve structure: *SHELXS2014* (Sheldrick, 2008 ▸); program(s) used to refine structure: *SHELXL2014* (Sheldrick, 2008 ▸); molecular graphics: *ORTEP-3 for Windows* (Farrugia, 2012 ▸); software used to prepare material for publication: *PLATON* (Spek, 2009 ▸).

## Supplementary Material

Crystal structure: contains datablock(s) global, I. DOI: 10.1107/S2056989014025857/hb7325sup1.cif

Structure factors: contains datablock(s) I. DOI: 10.1107/S2056989014025857/hb7325Isup2.hkl

Supporting information file. DOI: 10.1107/S2056989014025857/hb7325Isup3.cml

**Fig. 1.**
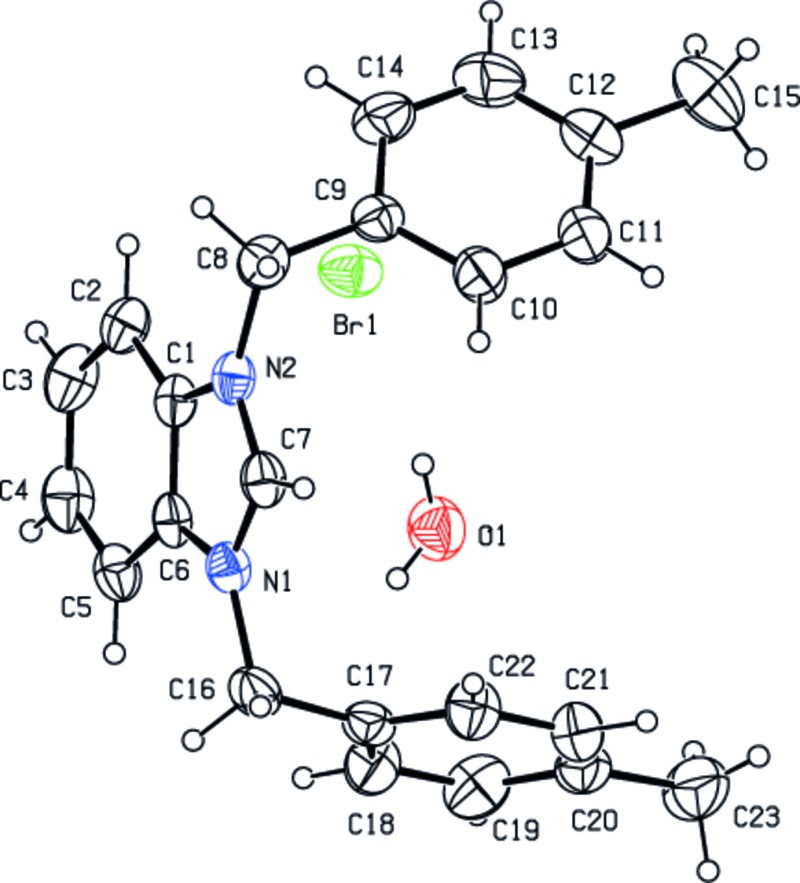
Perspective view of the molecular structure of the title compound with displacement ellipsoids drawn at the 30% probability level.

**Fig. 2.**
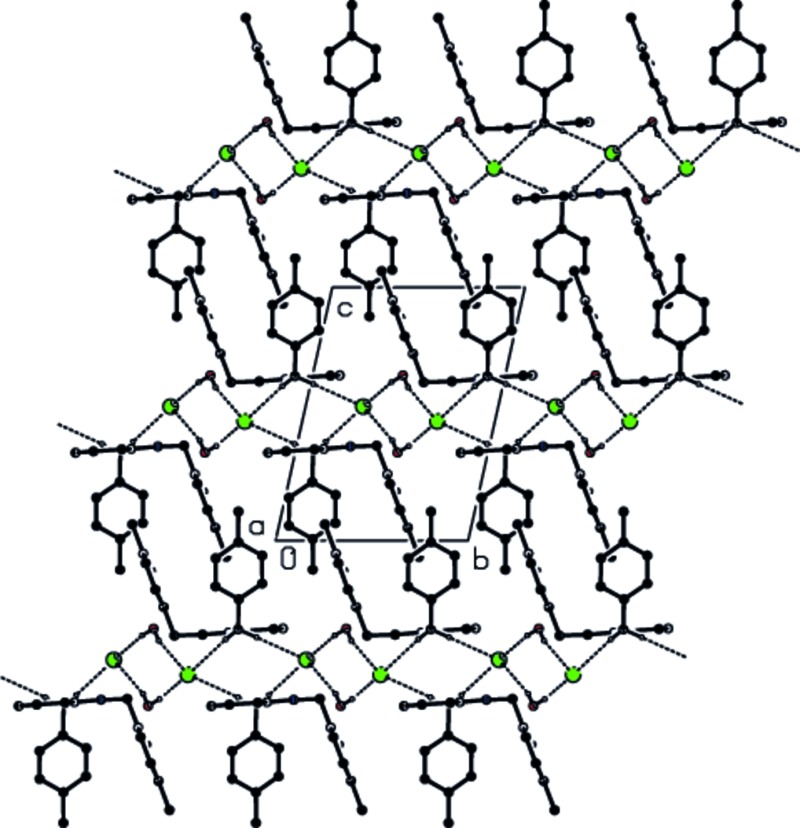
View of the hydrogen bonding and molecular packing of the title compound along a axis. Only H atoms involved in H bonding are shown.

. DOI: 10.1107/S2056989014025857/hb7325fig1.tif
Perspective view of the mol­ecular structure of the title compound with displacement ellipsoids drawn at the 30% probability level.

a . DOI: 10.1107/S2056989014025857/hb7325fig2.tif
View of the hydrogen bonding and mol­ecular packing of the title compound along *a* axis. Only H atoms involved in H bonding are shown.

CCDC reference: 1036037

Additional supporting information:  crystallographic information; 3D view; checkCIF report

## Figures and Tables

**Table 1:** Hydrogen-bond geometry (, )

*D*H*A*	*D*H	H*A*	*D* *A*	*D*H*A*
O1H1*W*Br1^i^	0.83(5)	2.47(5)	3.261(3)	162(5)
O1H2*W*Br1	0.82(5)	2.51(5)	3.318(3)	171(4)
C7H7O1	0.93	2.30	3.210(4)	166
C8H8*B*Br1	0.97	2.79	3.730(3)	163
C16H16*A*Br1^i^	0.97	2.91	3.875(4)	173
C16H16*B*Br1^ii^	0.97	2.79	3.741(4)	167

Symmetry codes: (i) 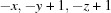; (ii) 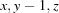.

## supplementary crystallographic information

### S1. Comment

N-heterocyclic carbenes (NHCs), on which many studies have been conducted over the past 40 years, have been frequently used as ligands in organometallic and coordination chemistry (Arduengo *et al.*, 1991; Akkoç & Gök, 2013; Akkoç *et al.* 2014; Berding *et al.*, 2009; Gök *et al.*, 2014). These ligands have such properties as being strong-donors, weak-acceptors, of low toxicity, easily-synthesized and able to control the steric and electronic effects of substituents on the nitrogen atom, and being more stable against air and moisture compared to phosphine types.

In the title compound (Fig. 1), the benzene rings (C9–C14 and C17–C22) which form a dihedral angle of 75.4 (2)° make dihedral angles of 73.18 (16) and 77.52 (16)° with respect to the central benzimidazole ring system (N1/N2/C1–C7). All bond lengths and bond angles in Table 1 are in normal range, and they are in a good agreement with those found in related compounds (Akkurt *et al.*, 2011; Akkurt *et al.*, 2012).

The crystal packing features C—H···O, O—H···Br and C—H···Br hydrogen bonds (Table 2, Fig. 2) together with π-π stacking interactions between the benzene and imidazolium rings (*Cg*1: C1–C6 and *Cg*2: N1/N2/C1/C6/C7) [*Cg*1···*Cg*2 (1 - *x*, -*y*, 1 - *z*) = 3.5401 (17) Å] and between the benzene rings of the benzimidazole ring system [*Cg*2···*Cg*2 (1 - *x*, -*y*, 1 - *z*) = 3.8815 (18) Å].

### S2. Experimental

To a solution of benzimidazole and potassium hidroxide in ethyl alcohol, 4-methylbenzyl bromide was added slowly. This mixture was refluxed at 18 h. Then, it was filtered and was dried under vacuum. 4-Methylbenzyl bromide (1.0 mmol) was added slowly to a solution of the obtained *N*-4-methylbenzylbenzimidazole (1.0 mmol) in DMF (4 ml) at room temperature and the resulting mixture was heated up to 353 K for 12 h. Diethyl ether (15 ml) was added to obtain a crystalline solid which was filtered off. The solid was washed with diethyl ether (2x15 ml) and dried under vacuum. The crude product was recrystallized from ethyl alcohol/diethyl ether at room temperature to yield colourless blocks.

### S3. Refinement

The H atoms H1W and H2W of the water molecule were located in a difference Fourier map. Their positions were refined with O—H = 0.82 (2) Å, but their temperature factors were refined isotropically with *U*_iso_(H) = 1.5*U*_eq_(O). H atoms attached to C atoms were placed in calculated positions with C—H = 0.93 - 0.97 Å, and refined using a riding model with *U*_iso_(H) = 1.2 or 1.5*U*_eq_(C). The highest peak and the deepest hole in the final difference Fourier map are located 0.97 Å from Br1 and 0.81 Å from Br1, respectively.

### Crystal data

**Table:**

C_23_H_23_N_2_^+^·Br^−^·H_2_O	*Z* = 2
*M**_r_* = 425.35	*F*(000) = 440
Triclinic, *P*1	*D*_x_ = 1.329 Mg m^−^^3^
Hall symbol: -P 1	Mo *K*α radiation, λ = 0.71073 Å
*a* = 9.3846 (3) Å	Cell parameters from 7459 reflections
*b* = 9.7174 (3) Å	θ = 2.3–27.0°
*c* = 12.5603 (4) Å	µ = 1.95 mm^−^^1^
α = 76.405 (2)°	*T* = 296 K
β = 84.739 (2)°	Plane, colourless
γ = 72.696 (2)°	0.15 × 0.10 × 0.06 mm
*V* = 1062.65 (6) Å^3^	

### Data collection

**Table:**

Bruker APEXII CCD diffractometer	3144 reflections with *I* > 2σ(*I*)
Radiation source: sealed tube	*R*_int_ = 0.035
Graphite monochromator	θ_max_ = 26.4°, θ_min_ = 1.7°
φ and ω scans	*h* = −11→11
22302 measured reflections	*k* = −12→12
4342 independent reflections	*l* = −15→15

### Refinement

**Table:**

Refinement on *F*^2^	2 restraints
Least-squares matrix: full	H atoms treated by a mixture of independent and constrained refinement
*R*[*F*^2^ > 2σ(*F*^2^)] = 0.048	*w* = 1/[σ^2^(*F*_o_^2^) + (0.0735*P*)^2^ + 0.2354*P*] where *P* = (*F*_o_^2^ + 2*F*_c_^2^)/3
*wR*(*F*^2^) = 0.135	(Δ/σ)_max_ < 0.001
*S* = 1.06	Δρ_max_ = 1.15 e Å^−^^3^
4342 reflections	Δρ_min_ = −0.49 e Å^−^^3^
252 parameters	

### Special details

**Table:**

Geometry. Bond distances, angles *etc*. have been calculated using the rounded fractional coordinates. All su's are estimated from the variances of the (full) variance-covariance matrix. The cell e.s.d.'s are taken into account in the estimation of distances, angles and torsion angles
Refinement. Refinement on *F*^2^ for ALL reflections except those flagged by the user for potential systematic errors. Weighted *R*-factors *wR* and all goodnesses of fit *S* are based on *F*^2^, conventional *R*-factors *R* are based on *F*, with *F* set to zero for negative *F*^2^. The observed criterion of *F*^2^ > σ(*F*^2^) is used only for calculating -*R*-factor-obs *etc*. and is not relevant to the choice of reflections for refinement. *R*-factors based on *F*^2^ are statistically about twice as large as those based on *F*, and *R*-factors based on ALL data will be even larger.

### Fractional atomic coordinates and isotropic or equivalent isotropic displacement parameters (Å^2^)

**Table:**

	*x*	*y*	*z*	*U*_iso_*/*U*_eq_	
Br1	0.15363 (4)	0.70096 (4)	0.47065 (3)	0.0746 (2)	
N1	0.2897 (3)	0.1364 (3)	0.36229 (18)	0.0512 (8)	
N2	0.4267 (3)	0.2795 (2)	0.37164 (18)	0.0501 (8)	
C1	0.5241 (3)	0.1441 (3)	0.3623 (2)	0.0474 (8)	
C2	0.6799 (4)	0.0955 (4)	0.3603 (3)	0.0597 (11)	
C3	0.7420 (4)	−0.0495 (4)	0.3526 (3)	0.0730 (12)	
C4	0.6544 (4)	−0.1407 (4)	0.3469 (3)	0.0714 (13)	
C5	0.5022 (4)	−0.0930 (3)	0.3487 (2)	0.0601 (10)	
C6	0.4383 (3)	0.0537 (3)	0.3562 (2)	0.0484 (9)	
C7	0.2888 (3)	0.2699 (3)	0.3712 (2)	0.0532 (9)	
C8	0.4673 (4)	0.4144 (3)	0.3749 (3)	0.0599 (11)	
C9	0.4849 (3)	0.5039 (3)	0.2621 (3)	0.0543 (10)	
C10	0.3662 (4)	0.5668 (4)	0.1937 (3)	0.0768 (14)	
C11	0.3829 (5)	0.6477 (4)	0.0897 (3)	0.0781 (12)	
C12	0.5159 (5)	0.6716 (4)	0.0512 (3)	0.0731 (14)	
C13	0.6324 (5)	0.6105 (6)	0.1209 (4)	0.0969 (17)	
C14	0.6195 (4)	0.5281 (5)	0.2240 (4)	0.0828 (16)	
C15	0.5316 (6)	0.7626 (6)	−0.0641 (4)	0.1146 (19)	
C16	0.1558 (4)	0.0910 (4)	0.3505 (3)	0.0629 (11)	
C17	0.1196 (3)	0.1227 (3)	0.2314 (3)	0.0552 (10)	
C18	0.1778 (4)	0.0197 (4)	0.1693 (3)	0.0744 (14)	
C19	0.1519 (5)	0.0524 (5)	0.0591 (3)	0.0863 (17)	
C20	0.0658 (4)	0.1912 (5)	0.0063 (3)	0.0724 (14)	
C21	0.0054 (5)	0.2899 (4)	0.0696 (3)	0.0795 (16)	
C22	0.0303 (4)	0.2586 (4)	0.1806 (3)	0.0724 (12)	
C23	0.0438 (6)	0.2267 (7)	−0.1146 (3)	0.110 (2)	
O1	−0.0190 (4)	0.5308 (3)	0.3474 (2)	0.0850 (11)	
H2	0.73790	0.15690	0.36400	0.0720*	
H3	0.84540	−0.08750	0.35110	0.0880*	
H4	0.70140	−0.23780	0.34160	0.0850*	
H5	0.44440	−0.15460	0.34530	0.0720*	
H7	0.20290	0.34660	0.37630	0.0640*	
H8A	0.56020	0.38660	0.41330	0.0720*	
H8B	0.39050	0.47440	0.41540	0.0720*	
H10	0.27300	0.55480	0.21780	0.0920*	
H11	0.30080	0.68710	0.04450	0.0940*	
H13	0.72460	0.62560	0.09750	0.1160*	
H14	0.70230	0.48810	0.26850	0.0990*	
H15A	0.49850	0.86580	−0.06310	0.1710*	
H15B	0.47190	0.74250	−0.11340	0.1710*	
H15C	0.63430	0.73670	−0.08800	0.1710*	
H16A	0.07180	0.14440	0.38980	0.0760*	
H16B	0.17390	−0.01380	0.38180	0.0760*	
H18	0.23590	−0.07390	0.20220	0.0890*	
H19	0.19270	−0.01960	0.01880	0.1030*	
H21	−0.05540	0.38250	0.03730	0.0950*	
H22	−0.01330	0.32970	0.22130	0.0870*	
H23A	−0.02220	0.17570	−0.13060	0.1660*	
H23B	0.13840	0.19600	−0.15140	0.1660*	
H23C	0.00120	0.33120	−0.13950	0.1660*	
H1W	−0.069 (5)	0.490 (6)	0.395 (4)	0.1280*	
H2W	0.017 (6)	0.582 (5)	0.374 (4)	0.1280*	

### Atomic displacement parameters (Å^2^)

**Table:**

	*U*^11^	*U*^22^	*U*^33^	*U*^12^	*U*^13^	*U*^23^
Br1	0.0632 (3)	0.0626 (2)	0.1010 (3)	−0.0181 (2)	0.0024 (2)	−0.0249 (2)
N1	0.0516 (14)	0.0507 (14)	0.0456 (13)	−0.0114 (11)	0.0027 (10)	−0.0053 (11)
N2	0.0516 (14)	0.0446 (13)	0.0468 (13)	−0.0021 (11)	−0.0055 (10)	−0.0095 (10)
C1	0.0533 (16)	0.0431 (14)	0.0398 (14)	−0.0032 (13)	−0.0048 (12)	−0.0097 (12)
C2	0.0523 (18)	0.0612 (19)	0.0595 (18)	−0.0036 (15)	−0.0088 (14)	−0.0147 (15)
C3	0.057 (2)	0.075 (2)	0.072 (2)	0.0122 (18)	−0.0080 (16)	−0.0248 (18)
C4	0.082 (3)	0.0510 (18)	0.067 (2)	0.0066 (18)	−0.0042 (18)	−0.0176 (16)
C5	0.076 (2)	0.0452 (16)	0.0528 (18)	−0.0091 (15)	−0.0026 (15)	−0.0087 (13)
C6	0.0527 (16)	0.0470 (15)	0.0345 (14)	−0.0045 (13)	0.0010 (12)	−0.0009 (12)
C7	0.0515 (17)	0.0480 (16)	0.0491 (16)	−0.0014 (13)	0.0014 (13)	−0.0071 (13)
C8	0.0619 (19)	0.0480 (16)	0.069 (2)	−0.0058 (14)	−0.0113 (15)	−0.0205 (15)
C9	0.0537 (17)	0.0420 (15)	0.069 (2)	−0.0103 (13)	−0.0042 (14)	−0.0187 (14)
C10	0.0524 (19)	0.074 (2)	0.089 (3)	−0.0164 (17)	−0.0051 (17)	0.010 (2)
C11	0.073 (2)	0.074 (2)	0.079 (2)	−0.0242 (19)	−0.0078 (19)	0.006 (2)
C12	0.079 (3)	0.058 (2)	0.083 (2)	−0.0247 (18)	0.017 (2)	−0.0174 (18)
C13	0.072 (3)	0.115 (3)	0.111 (3)	−0.051 (3)	0.018 (2)	−0.015 (3)
C14	0.057 (2)	0.092 (3)	0.103 (3)	−0.027 (2)	−0.012 (2)	−0.017 (2)
C15	0.140 (4)	0.101 (3)	0.096 (3)	−0.049 (3)	0.037 (3)	−0.005 (3)
C16	0.0608 (19)	0.066 (2)	0.060 (2)	−0.0262 (16)	0.0099 (15)	−0.0038 (16)
C17	0.0481 (16)	0.0573 (18)	0.0625 (19)	−0.0224 (14)	0.0055 (14)	−0.0104 (15)
C18	0.080 (2)	0.060 (2)	0.081 (3)	−0.0076 (18)	−0.0144 (19)	−0.0222 (18)
C19	0.086 (3)	0.095 (3)	0.085 (3)	−0.017 (2)	−0.001 (2)	−0.045 (2)
C20	0.068 (2)	0.092 (3)	0.065 (2)	−0.039 (2)	−0.0019 (17)	−0.011 (2)
C21	0.084 (3)	0.067 (2)	0.081 (3)	−0.020 (2)	−0.017 (2)	0.000 (2)
C22	0.073 (2)	0.064 (2)	0.074 (2)	−0.0070 (18)	−0.0038 (18)	−0.0185 (18)
C23	0.112 (4)	0.165 (5)	0.069 (3)	−0.072 (3)	−0.004 (2)	−0.012 (3)
O1	0.102 (2)	0.0815 (19)	0.0707 (17)	−0.0267 (15)	−0.0048 (14)	−0.0135 (14)

### Geometric parameters (Å, º)

**Table:**

N1—C6	1.393 (4)	C21—C22	1.382 (5)
N1—C7	1.326 (4)	C2—H2	0.9300
N1—C16	1.480 (5)	C3—H3	0.9300
N2—C1	1.385 (4)	C4—H4	0.9300
N2—C7	1.326 (4)	C5—H5	0.9300
N2—C8	1.480 (4)	C7—H7	0.9300
C1—C2	1.396 (5)	C8—H8A	0.9700
C1—C6	1.375 (4)	C8—H8B	0.9700
C2—C3	1.378 (5)	C10—H10	0.9300
C3—C4	1.393 (5)	C11—H11	0.9300
C4—C5	1.364 (5)	C13—H13	0.9300
C5—C6	1.395 (4)	C14—H14	0.9300
C8—C9	1.501 (5)	C15—H15A	0.9600
C9—C10	1.370 (5)	C15—H15B	0.9600
C9—C14	1.376 (5)	C15—H15C	0.9600
C10—C11	1.379 (5)	C16—H16A	0.9700
C11—C12	1.362 (7)	C16—H16B	0.9700
C12—C13	1.364 (7)	C18—H18	0.9300
C12—C15	1.529 (6)	C19—H19	0.9300
C13—C14	1.369 (7)	C21—H21	0.9300
C16—C17	1.504 (5)	C22—H22	0.9300
C17—C18	1.368 (5)	C23—H23A	0.9600
C17—C22	1.377 (5)	C23—H23B	0.9600
C18—C19	1.373 (5)	C23—H23C	0.9600
C19—C20	1.393 (6)	O1—H1W	0.83 (5)
C20—C21	1.351 (6)	O1—H2W	0.82 (5)
C20—C23	1.496 (5)		
			
C6—N1—C7	107.4 (3)	C5—C4—H4	119.00
C6—N1—C16	127.1 (3)	C4—C5—H5	122.00
C7—N1—C16	125.2 (3)	C6—C5—H5	122.00
C1—N2—C7	107.8 (2)	N1—C7—H7	125.00
C1—N2—C8	126.7 (3)	N2—C7—H7	125.00
C7—N2—C8	125.5 (2)	N2—C8—H8A	109.00
N2—C1—C2	130.8 (3)	N2—C8—H8B	109.00
N2—C1—C6	107.0 (3)	C9—C8—H8A	109.00
C2—C1—C6	122.3 (3)	C9—C8—H8B	109.00
C1—C2—C3	115.6 (3)	H8A—C8—H8B	108.00
C2—C3—C4	121.9 (4)	C9—C10—H10	120.00
C3—C4—C5	122.5 (3)	C11—C10—H10	120.00
C4—C5—C6	116.1 (3)	C10—C11—H11	119.00
N1—C6—C1	106.9 (3)	C12—C11—H11	119.00
N1—C6—C5	131.3 (3)	C12—C13—H13	119.00
C1—C6—C5	121.8 (3)	C14—C13—H13	119.00
N1—C7—N2	110.9 (3)	C9—C14—H14	120.00
N2—C8—C9	112.0 (3)	C13—C14—H14	120.00
C8—C9—C10	121.0 (3)	C12—C15—H15A	109.00
C8—C9—C14	121.6 (3)	C12—C15—H15B	109.00
C10—C9—C14	117.4 (4)	C12—C15—H15C	109.00
C9—C10—C11	120.9 (4)	H15A—C15—H15B	110.00
C10—C11—C12	122.0 (4)	H15A—C15—H15C	109.00
C11—C12—C13	116.4 (4)	H15B—C15—H15C	110.00
C11—C12—C15	121.1 (4)	N1—C16—H16A	110.00
C13—C12—C15	122.5 (4)	N1—C16—H16B	110.00
C12—C13—C14	122.8 (4)	C17—C16—H16A	110.00
C9—C14—C13	120.4 (4)	C17—C16—H16B	110.00
N1—C16—C17	110.1 (3)	H16A—C16—H16B	108.00
C16—C17—C18	121.3 (3)	C17—C18—H18	120.00
C16—C17—C22	120.8 (3)	C19—C18—H18	119.00
C18—C17—C22	117.9 (3)	C18—C19—H19	119.00
C17—C18—C19	121.0 (4)	C20—C19—H19	119.00
C18—C19—C20	121.5 (4)	C20—C21—H21	119.00
C19—C20—C21	116.7 (3)	C22—C21—H21	119.00
C19—C20—C23	120.7 (4)	C17—C22—H22	120.00
C21—C20—C23	122.7 (4)	C21—C22—H22	120.00
C20—C21—C22	122.5 (4)	C20—C23—H23A	110.00
C17—C22—C21	120.4 (3)	C20—C23—H23B	109.00
C1—C2—H2	122.00	C20—C23—H23C	109.00
C3—C2—H2	122.00	H23A—C23—H23B	109.00
C2—C3—H3	119.00	H23A—C23—H23C	109.00
C4—C3—H3	119.00	H23B—C23—H23C	109.00
C3—C4—H4	119.00	H1W—O1—H2W	109 (5)
			
C7—N1—C6—C1	0.2 (3)	C4—C5—C6—C1	−0.5 (4)
C16—N1—C6—C1	174.8 (3)	N2—C8—C9—C14	−117.1 (4)
C7—N1—C6—C5	178.4 (3)	N2—C8—C9—C10	64.0 (4)
C16—N1—C6—C5	−7.0 (5)	C8—C9—C14—C13	−179.8 (4)
C6—N1—C7—N2	−0.2 (3)	C8—C9—C10—C11	−179.3 (3)
C16—N1—C7—N2	−174.9 (3)	C14—C9—C10—C11	1.7 (5)
C6—N1—C16—C17	−85.5 (4)	C10—C9—C14—C13	−0.9 (6)
C7—N1—C16—C17	88.2 (3)	C9—C10—C11—C12	−1.5 (6)
C1—N2—C8—C9	86.2 (3)	C10—C11—C12—C15	−179.6 (4)
C7—N2—C8—C9	−89.9 (3)	C10—C11—C12—C13	0.3 (6)
C7—N2—C1—C6	0.1 (3)	C11—C12—C13—C14	0.6 (7)
C8—N2—C1—C6	−176.6 (3)	C15—C12—C13—C14	−179.6 (5)
C7—N2—C1—C2	−179.0 (3)	C12—C13—C14—C9	−0.3 (8)
C8—N2—C1—C2	4.4 (5)	N1—C16—C17—C18	90.4 (4)
C1—N2—C7—N1	0.1 (3)	N1—C16—C17—C22	−87.5 (4)
C8—N2—C7—N1	176.8 (3)	C16—C17—C18—C19	−175.9 (4)
N2—C1—C2—C3	178.5 (3)	C22—C17—C18—C19	2.1 (6)
C2—C1—C6—C5	0.6 (4)	C16—C17—C22—C21	175.9 (4)
N2—C1—C6—C5	−178.5 (2)	C18—C17—C22—C21	−2.1 (5)
C6—C1—C2—C3	−0.5 (5)	C17—C18—C19—C20	0.1 (7)
C2—C1—C6—N1	179.0 (3)	C18—C19—C20—C21	−2.1 (7)
N2—C1—C6—N1	−0.2 (3)	C18—C19—C20—C23	177.6 (4)
C1—C2—C3—C4	0.2 (5)	C19—C20—C21—C22	2.0 (7)
C2—C3—C4—C5	−0.1 (6)	C23—C20—C21—C22	−177.6 (4)
C3—C4—C5—C6	0.2 (5)	C20—C21—C22—C17	0.0 (7)
C4—C5—C6—N1	−178.4 (3)		

### Hydrogen-bond geometry (Å, º)

**Table:**

*D*—H···*A*	*D*—H	H···*A*	*D*···*A*	*D*—H···*A*
O1—H1*W*···Br1^i^	0.83 (5)	2.47 (5)	3.261 (3)	162 (5)
O1—H2*W*···Br1	0.82 (5)	2.51 (5)	3.318 (3)	171 (4)
C7—H7···O1	0.93	2.30	3.210 (4)	166
C8—H8*B*···Br1	0.97	2.79	3.730 (3)	163
C16—H16*A*···Br1^i^	0.97	2.91	3.875 (4)	173
C16—H16*B*···Br1^ii^	0.97	2.79	3.741 (4)	167

Symmetry codes: (i) −*x*, −*y*+1, −*z*+1; (ii) *x*, *y*−1, *z*.
