# Supplementary figures and images for: Crystal structure of 1,3-bis­(4-methyl­benz­yl)-1H-1,3-benzimidazol-3-ium bromide monohydrate
Source: Acta Crystallogr E Crystallogr Commun. 2015 Jan 1;71(Pt 1):o10–1. doi: 10.1107/S2056989014025857 (PMC4331869; doi:10.1107/S2056989014025857)

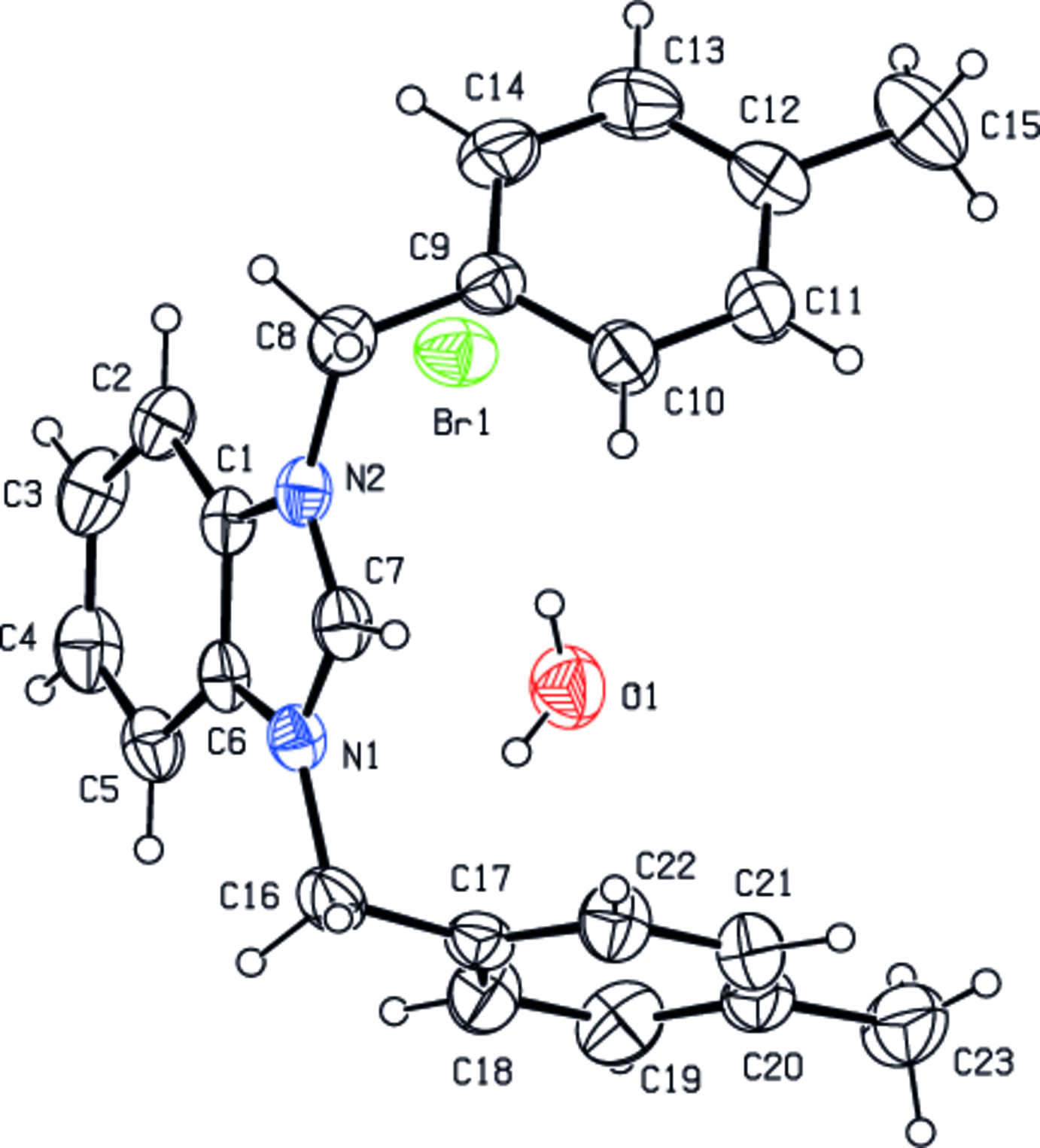

Supplement: Supplementary file 4 [file e-71-00o10-fig1.tif]

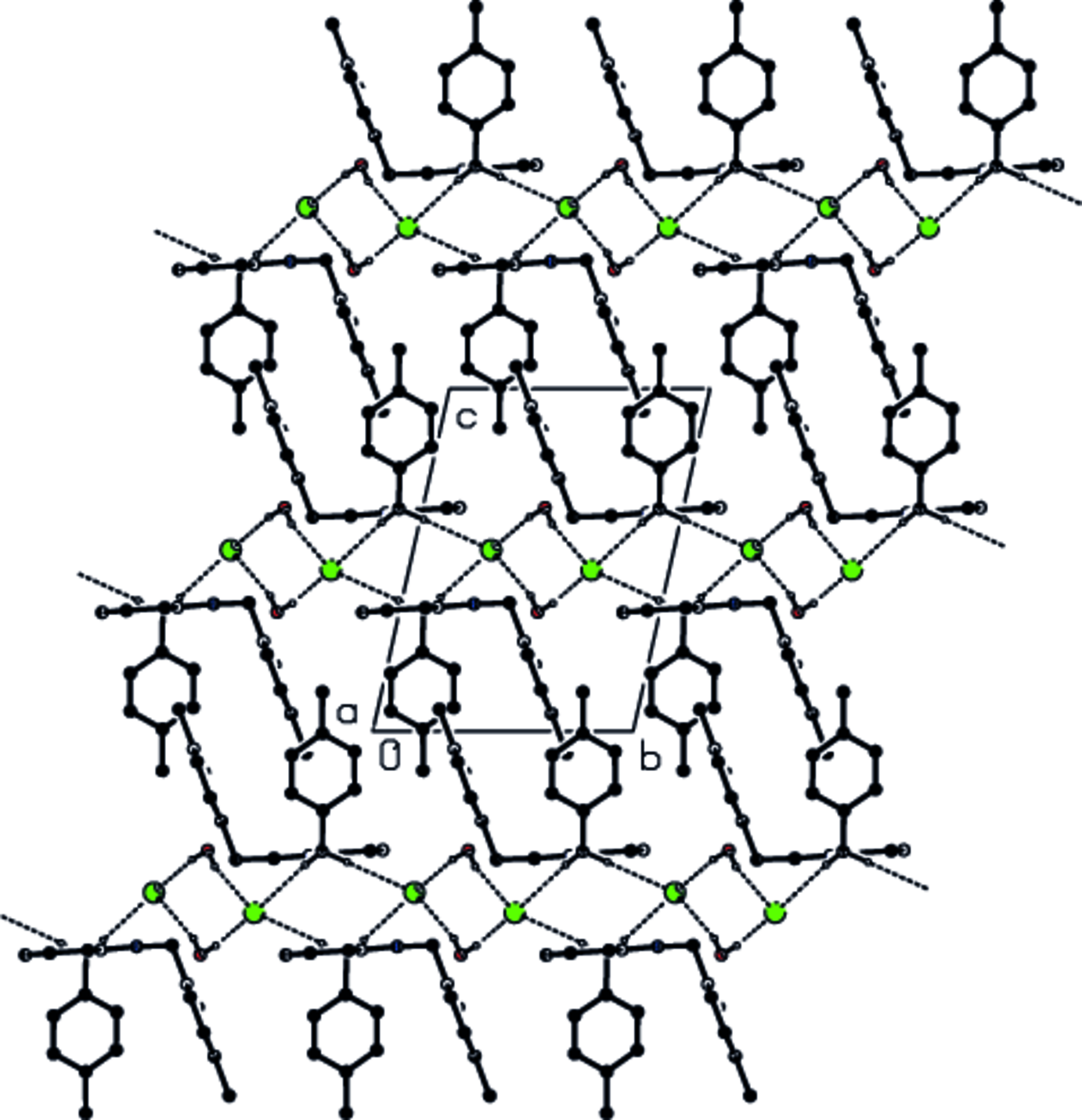

Supplement: Supplementary file 5 [file e-71-00o10-fig2.tif]
